# The Human G Protein-Coupled ATP Receptor P2Y_11_ Is Associated With IL-10 Driven Macrophage Differentiation

**DOI:** 10.3389/fimmu.2019.01870

**Published:** 2019-08-09

**Authors:** Georg Gruenbacher, Hubert Gander, Andrea Rahm, Gabriele Dobler, Astrid Drasche, Jakob Troppmair, Walter Nussbaumer, Martin Thurnher

**Affiliations:** ^1^Immunotherapy Unit, Department of Urology, Medical University of Innsbruck, Innsbruck, Austria; ^2^Daniel Swarovski Research Laboratory, Department of Visceral, Transplant and Thoracic Surgery, Medical University of Innsbruck, Innsbruck, Austria; ^3^Central Institute for Blood Transfusion and Immunology, Medical University Hospital Innsbruck, Innsbruck, Austria

**Keywords:** P2Y_11_, ATP, NAD^+^, IL-10, M2 macrophages

## Abstract

The G protein-coupled P2Y_11_ receptor is known to sense extracellular ATP during inflammatory and immune responses. The dinucleotide NAD^+^ has also been proposed to be a P2Y_11_ receptor ligand but its role is less clear. Here, we have examined for the first time human P2Y_11_ receptor protein levels and show that the receptor was upregulated during polarization of M2 macrophages. IL-10 reinforced P2Y_11_ receptor expression during differentiation of M2c macrophages expressing CD163, CD16, and CD274 (PD-L1). Nutlin-3a mediated p53 stabilization further increased P2Y_11_ receptor, CD16, and PD-L1 expression. AMP-activated kinase (AMPK), which mediates anti-inflammatory effects of IL-10, and nicotinamide phosphoribosyltransferase (NAMPT), the rate-limiting enzyme of the NAD^+^ salvage pathway, which is under the control of AMPK, were also required for P2Y_11_ receptor expression. The P2Y_11_ receptor agonist ATPγS and NAD^+^ could independently stimulate the production of IL-8 in M2 macrophages, however, only the ATPγS-induced response was mediated by P2Y_11_ receptor. Both in a recombinant system and in macrophages, P2Y_11_ receptor-driven IL-8 production predominantly depended on IkB kinase (IKK), and extracellular signal–regulated kinase (ERK). In conclusion, our data indicate that an AMPK-NAMPT-NAD^+^ signaling axis promotes P2Y_11_ receptor expression during M2 polarization of human macrophages in response to IL-10. PD-L1 expressing M2c macrophages that secrete the cancer-promoting chemokine IL-8 in response to P2Y_11_ receptor stimulation may represent an important target in checkpoint blockade immunotherapy.

## Introduction

P2Y_11_ receptor is an unconventional member of the P2Y family of G protein-coupled receptors (GPCR), which currently comprises eight members (P2Y_1_, P2Y_2_, P2Y_4_, P2Y_6_, P2Y_11_, P2Y_12_, P2Y_13_, and P2Y_14_) (1, 2). The P2Y_11_ receptor, which couples to both phospholipase C (PLC), and adenylyl cyclase (AC), is preferentially activated by ATP (1). The dinucleotide NAD^+^, another key regulator of metabolic and inflammatory processes (3, 4), has also been considered an agonist at the human P2Y_11_ receptor (5), but its role is less clear. Human P2RY11 has first been cloned more than two decades ago (6). However, the role of P2Y_11_ receptor has been difficult to assess due to its apparent absence in rodents and due to the limited range of specific pharmacological, and biochemical tools (1). In earlier studies, P2Y_11_ receptor involvement was suggested through detection of P2Y_11_ receptor-encoding mRNA, by exclusion of other possible ATP receptors, and later by siRNA knockdown of the receptor to support pharmacological data, often related to cell survival and cytokine production as well as cell migration and differentiation (7–11). Functional P2Y_11_ receptor expression has been reported in innate immune cells (1, 2) such as dendritic cells (DCs) (8, 9) and macrophages (12) as well as in adaptive immune cells (7, 13), strongly supporting its relevance in immune regulation.

Blood monocytes can differentiate into DCs or macrophages. Moreover, the microenvironment governs the development of macrophage subsets with distinct functional phenotypes (14). Two contrasting and cross-regulating effector phenotypes, known as classically activated macrophages (M1 macrophages), and alternatively activated macrophages (M2 macrophages), have been defined. Moreover, M2 macrophages may be further subdivided into M2a, M2b, and M2c cells (15). While interleukin-4 (IL-4) promotes the development of M2a, IL-10 is critical for M2c macrophage generation. IL-4 not only promotes M2a macrophages but also cooperates with granulocyte/macrophage colony-stimulating factor (GM-CSF) to drive DC differentiation from monocytes *in vitro* (16, 17). The conserved serine/threonine kinase AMPK has been shown to be required for the IL-10-mediated activation of anti-inflammatory pathways during macrophage polarization (18–21). AMPK in turn can cause an increase in cellular NAD^+^ levels via the salvage pathway of NAD^+^ synthesis (22).

In the present work, we have examined P2Y_11_ receptor expression and function during monocyte differentiation toward DCs and macrophage subsets. We show that P2Y_11_ receptor expression was particularly upregulated during IL-10 driven M2c macrophage differentiation via AMPK, NAMPT, and NAD^+^ metabolism. Consistent with its role in metabolic regulation (23), p53 was also found to promote P2Y_11_ receptor expression.

## Materials and Methods

### Reagents

P2Y_11_ receptor agonists and controls: NF546 (TOCRIS), ATPγS, ATP, ADP, AMP, UTP, NAD^+^ (all from Sigma Aldrich); P2Y_11_ receptor inhibitors: NF340, NF157 (TOCRIS); Compound C (AMPK inhibitor) was from Enzo Life Sciences; EX-527 (SIRT1 inhibitor), FK-866 (NAMPT inhibitor), NMN (NAD^+^ precursor), nutlin-3a (MDM2 inhibitor/p53 stabilizer), TPCA-1 (IKK inhibitor), and U0126 (MEK/ERK inhibitor) were from Sigma.

### Monocyte Isolation and Differentiation

Inclusion of healthy donors was approved by the local institutional review board. Buffy coats were obtained after written informed consent and provided by the Central Institute for Blood Transfusion (Innsbruck, Austria). PBMCs were isolated from these samples by density gradient centrifugation (Lymphoprep). PBMCs were depleted of CD16^+^ cells using CD16 microbeads and LD columns (Miltenyi Biotec). Monocytes were then isolated using CD14 microbeads and LS columns (purity >95%). Monocyte-derived DCs were generated using GM-CSF and IL-4 as described previously (24). Monocytes were differentiated toward M2a macrophages for 5 days in the presence of recombinant M-CSF (50 ng/ml) and IL-4 (800 U/ml) or toward M2c in the presence of recombinant M-CSF (50 ng/ml) and IL-10 (10 ng/ml) (25).

### Phagocytosis of Latex Beads

Latex beads (diameter, 4 μm; Thermo Fisher Scientific) were centrifuged (1 min at 100 g) onto adherent day-5 M2c macrophages at a ratio of 10:1 to synchronize binding and internalization. After 1 h at 37°C, non-adherent beads were removed with cold PBS and cells were immediately photographed by phase contrast microscopy (Olympus CK2, magnification: 200-fold).

### Ectopic P2Y_11_ Expression and CRISPR/Cas9-Mediated Knockdown

The glioma cell line 1321N1 (ECACC 86030402), a grade II human brain astrocytoma devoid of functional P2X and P2Y receptors, was used as a host cell line for ectopic expression of human P2Y_11_ receptor (26). In this expression system, the cDNA coding for the human P2Y_11_ receptor was cloned as an EcoRI-XbaI fragment into the bicistronic pEFIN3 vector for constitutive expression from an EF1alpha promoter, with the neomycin gene (conferring G418 resistance) being driven by an IRES sequence placed immediately after the P2Y_11_ receptor-coding cDNA (Perkin Elmer) (27, 28). Transfected cells were grown at 37°C in a humidified 5% CO_2_ atmosphere in Dulbecco's modified Eagle's medium (DMEM) supplemented with 10% (v/v) FBS, 1 mM sodium pyruvate, 2 mM L-alanyl-L-glutamine (Glutamax), 100 units/ml of penicillin, and 100 μg/ml of streptomycin. G418 (geneticin) was used at 400 μg/ml to select for stable transfectants.

For targeting P2RY11 with CRISPR/Cas9, we used reagents from Thermo Fisher Scientific (A35509). The following crRNAs were used: P2RY11 specific crRNA: CTGCCGACGACAAACTCAGT (CRISPR926511_CR; Position 254–273; NM_002566.4) and TGCTCAACGTGGATGCTCGG (CRISPR1125182_CR; Position 1007–1026; NM_002566.4);

Target-specific crRNA and tracrRNA (Invitrogen; A35507) were fused to form the functional guide RNA (gRNA). Cas9 protein (TrueCut Cas9 Protein v2; A36497) and gRNA were co-transfected into the target cell line using Lipofectamine CRISPRMAX Cas9 Transfection Reagent (Invitrogen; CMAX00003) followed by selection with G418-BC (500 μg/ml) for 8 days. P2RY11 knockdown was verified by flow cytometry and P2Y_11_-negative cells were sorted on a BD FACSAria at the local FACS core facility.

### Flow Cytometry

The following antibodies were used for macrophage phenotyping: rabbit polyclonal IgG anti-human P2Y_11_ receptor (THP, bs-1207R-A488), mouse IgG2b anti-human CD14 (BD Bioscience, clone MϕP9, 345787-APC), mouse IgG1k anti-human CD16 (BD Bioscience, clone B73.1, 332779-PE), mouse IgG1k anti-human CD163 (BD Bioscience, clone GHI/61, 556018-PE), mouse IgG1k anti-human CD274 (BD Bioscience, clone MIH1, 563738-PE).

Alexa Fluor 647-conjugated IgG1 (R&D clone # 505214) was used to detect human P2Y_11_ receptor on glioma cells after cell detachment using Accutase (Sigma-Aldrich). Cells were washed and then stained on ice for 30 min in the dark in PBS containing 0.5% FCS and 50 μg/ml human IgG (Octapharma) to block Fc γ receptors. Fixable viability dye eFluor 780 (Thermo Fisher Scientific) was used to label dead cells. For all samples, acquisition and analysis was performed on a FACSCanto II flow cytometer and FACS Diva 6.1.2 as well as FlowJo V7.2.5 software (BD Biosciences) by applying dead cell and doublet discrimination.

### IL-8 Measurement

IL-8 levels in supernatants were assessed at the indicated time points using the human BD CBA IL-8 Flex Set, BD Biosciences). Samples were analyzed with a FACSCanto II system and FCAP Array 1.0.1 software from BD Biosciences.

### Statistics

Results are expressed as mean ± SEM. The following tests were used: Student *t*-test for comparing the means of two groups and ANOVA for comparing means of more than two groups; an output of *p* < 0.05 was accepted as significantly different in all tests.

## Results

### P2Y_11_ Receptor Expression Is Upregulated During Macrophage Differentiation

**Figure 1:**
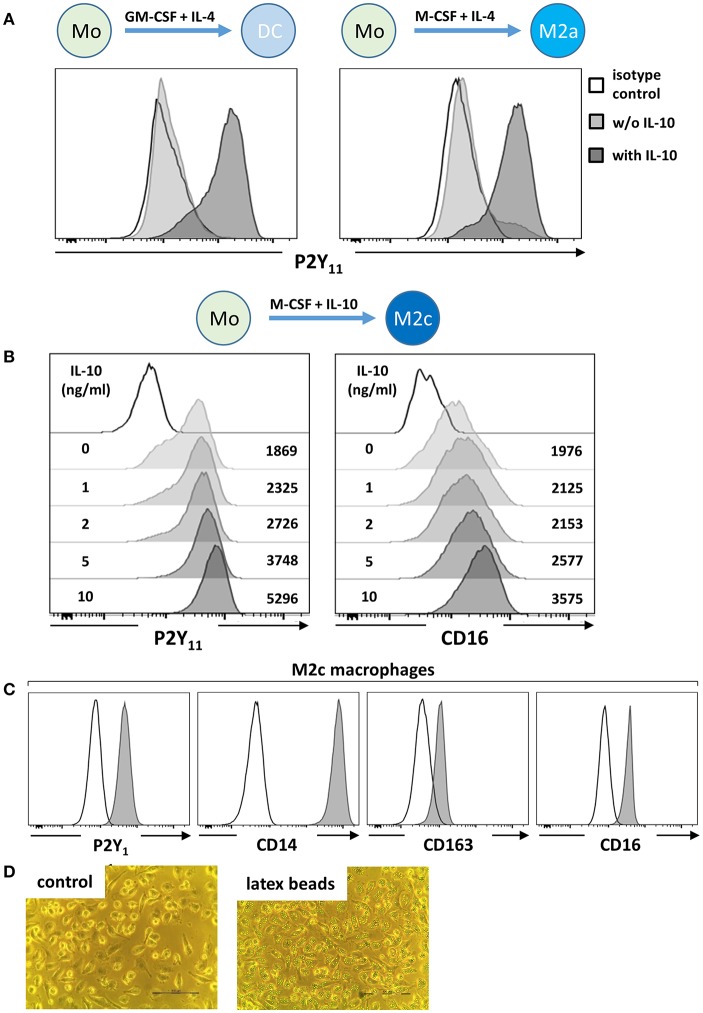
IL-10 promotes P2Y_11_ receptor surface expression during macrophage differentiation. **(A)** CD16-depleted human monocytes (CD16^−^CD14^+^) were cultured for 5 d with GM-CSF and IL-4 (DC protocol) or with M-CSF and IL-4 (M2a protocol), in the presence or absence of IL-10 (10 ng/ml). P2Y_11_ receptor surface expression was measured by flow cytometry. **(B)** CD16^−^CD14^+^ monocytes were cultured for 5 d with M-CSF and graded doses of IL-10 (M2c protocol). P2Y_11_ receptor and CD16 surface expression were measured by flow cytometry. Numbers are mean fluorescence intensities. **(C)** CD16^−^CD14^+^ monocytes were cultured for 5 d with M-CSF (50 ng/ml) and IL-10 (10 ng/ml) (M2c protocol). Cell surface expression of P2Y_11_ receptor, CD14, CD163, and CD16 was measured by flow cytometry. **(D)** Phagocytosis of latex beads by M2c macrophages; *n* = 6 from three independent donors.

Human monocyte-derived DCs (moDCs) have previously been suggested to express functional P2Y_11_ (8–11). When we examined moDCs by flow cytometry, we detected only low levels of P2Y_11_ receptor surface expression (Figure 1A), explaining the requirement for high agonist dosage in earlier studies (8, 9) and raising the question whether another monocyte-derived cell type might more substantially express P2Y_11_ receptor. From our own previous studies of moDCs we knew that M-CSF production was induced by GM-CSF in moDC cultures (24). We therefore differentiated monocytes with M-CSF and IL-4, a protocol that favors development of M2a macrophages (14), but P2Y_11_ receptor expression remained low (Figure 1A). We previously observed that IL-10 addition to moDC cultures stimulated c-fms (M-CSF receptor) expression on DCs, which thus acquired sensitivity to endogenous M-CSF and then deviated from the DC pathway and differentiated toward CD16-expressing macrophages (24). In our present re-examination, addition of IL-10 indeed induced strong P2Y_11_ receptor upregulation, both in moDC, and M2a cultures (Figure 1A), suggesting that P2Y_11_ receptor is associated with macrophage differentiation.

### IL-10 Promotes P2Y_11_ Receptor Upregulation During M2c Macrophage Differentiation

As a next step, we systematically investigated M-CSF-driven and IL-10 enhanced monocyte-to-macrophage differentiation (25), a condition that promotes M2c macrophage development (14). Monocytes cultured for 5 days with M-CSF alone (M2) already displayed substantial P2Y_11_ receptor expression (Figure 1B), also indicating that IL-4 present in moDC and M2a cultures had attenuated M-CSF-induced P2Y_11_ receptor upregulation (Figure 1A). In the M2c setting, addition of IL-10 substantially enhanced P2Y_11_ receptor and CD16 expression in a dose-dependent manner (Figure 1B). These findings indicated on the one hand that IL-10 was the driving force for P2Y_11_ receptor expression during M2c macrophage differentiation, and on the other that IL-10 can overcome the P2Y_11_ receptor-suppressing effects of IL-4 (Figure 1A). In addition to very high levels of CD14, these P2Y_11_high macrophages expressed the M2c-specific marker CD163 (hemoglobin-haptoglobin scavenger receptor) and CD16 (FcγRIII) (29, 30) (Figure 1C). Especially, CD163, and CD16 have been considered specific markers indicative of exposure to M-CSF and IL-10 (i.e., M2c polarization). We next addressed the more specialized M2c macrophage function of phagocytosis, which has previously been shown to rely on the combined action of M-CSF and IL-10 (29). Within 1 h of exposure to latex beads (4 μm in diameter), adherent M2c macrophages showed substantial phagocytosis, with most cells having engulfed over six beads (Figure 1D and Figure S1).

### Activation of p53 Further Increases P2Y_11_ Receptor Expression on M2c Macrophages

**Figure 2:**
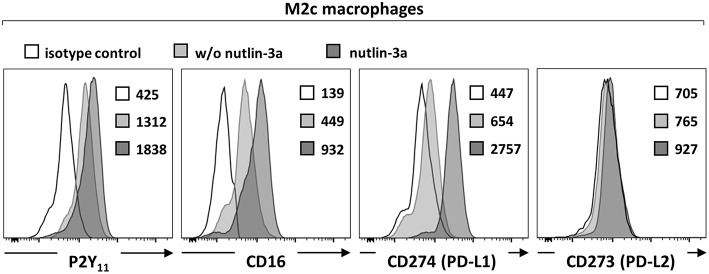
p53 activation increases P2Y_11_ receptor expression on M2c macrophages. CD16^−^CD14^+^ monocytes were cultured for 4 d with M-CSF and IL-10 (M2c protocol) and for additional 3 d with nutlin-3a (10 μM). P2Y_11_ receptor, CD16, and PD-L1/2 surface expression were measured by flow cytometry. Numbers are mean fluorescence intensities, *n* = 4 from two independent donors.

Macrophages have been shown to exhibit endogenous p53 activity, which increases during M2 polarization (31). The promoter region of human P2RY11 was previously found to contain three p53 noncanonical response elements (32) suggesting that stress-activated p53 regulates P2RY11 expression. To test p53 functionality and its potential effect on P2RY11 expression in M2c macrophages, we used nutlin-3a to stabilize, and thus activate wild-type p53. Nutlin-3a caused a further increase in P2Y_11_ receptor expression as well as a significant enhancement of CD16 expression (Figure 2). In accordance with previous findings (33), M2c macrophages induced by IL-10 expressed PD-L1 but not PD-L2 (Figure 2). Importantly, nutlin-3a also strongly enhanced PD-L1 expression in M2c macrophages (Figure 2). However, nutlin-3a could not induce PD-L2 expression.

### An AMPK-NAMPT-NAD^+^ Signaling Axis Is Required for P2Y_11_ Receptor Upregulation During M2c Macrophage Differentiation

**Figure 3:**
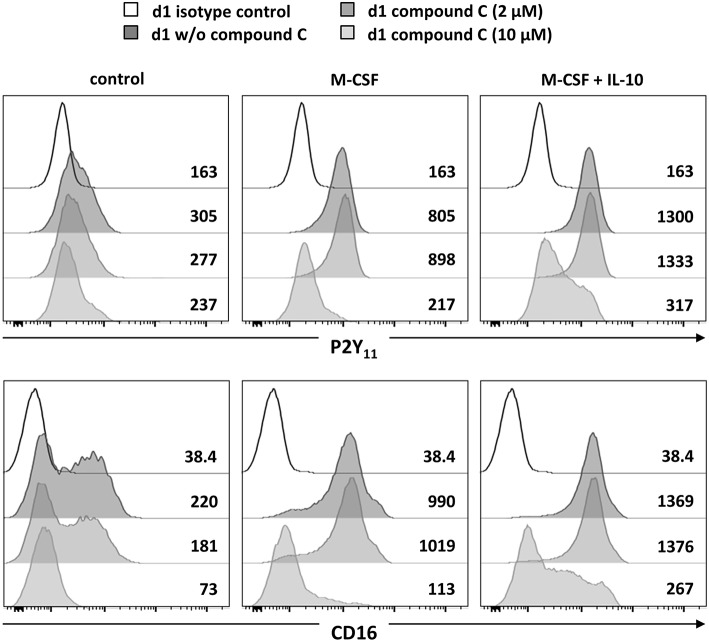
P2Y_11_ upregulation during M2c macrophage differentiation depends on AMPK, NAMPT, and NAD^+^. CD16^−^CD14^+^ monocytes were left untreated (control) or stimulated overnight with M-CSF alone or in combination with IL-10 in the presence or absence of the AMPK inhibitor compound C. P2Y_11_ receptor and CD16 surface expression were measured by flow cytometry. Numbers are mean fluorescence intensities.

**Figure 4:**
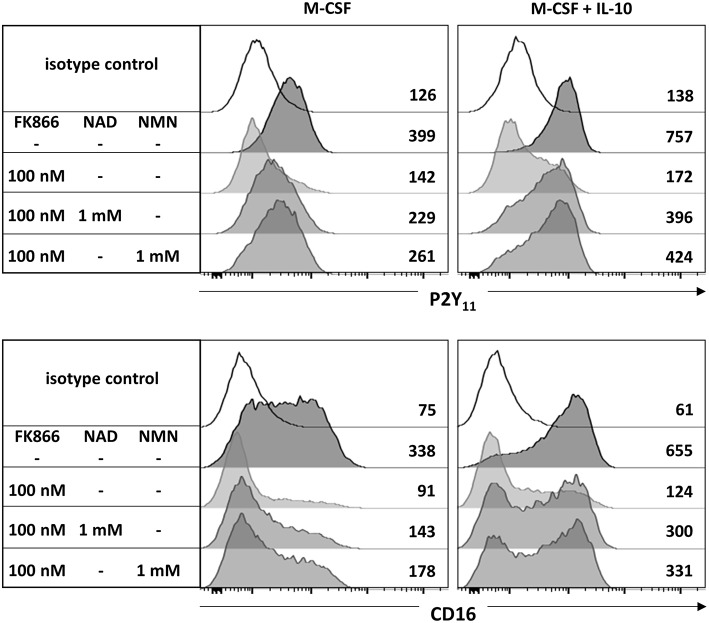
CD16^−^CD14^+^ monocytes were stimulated overnight with M-CSF alone or in combination with IL-10 in the presence or absence of the NAMPT inhibitor FK-866. NAD^+^ or its immediate precursor NMN were added at 1 mM to replenish intracellular NAD^+^ pools during NAMPT inhibition. P2Y_11_ receptor surface expression was measured by flow cytometry. Numbers are mean fluorescence intensities. *n* = 6 from three independent donors.

Since AMPK is known to inhibit DC activation (34) and to promote monocyte-to-macrophage differentiation in response to M-CSF and IL-10 (18, 20), we subsequently investigated the role of AMPK in the regulation of P2Y_11_ expression. In overnight stimulations with M-CSF alone or in combination with IL-10, Compound C, a well-established AMPK inhibitor (also known as dorsomorphin or BML-275), effectively prevented upregulation of P2Y_11_ receptor and also inhibited CD16 upregulation (Figure 3). AMPK activation is known to raise the intracellular levels of NAD^+^ (22). This increase in intracellular NAD^+^ can be mediated via posttranslational elevation of NAMPT (35), a key enzyme in the salvage pathway of NAD^+^ biosynthesis (3). NAMPT converts nicotinamide into nicotinamide mononucleotide (NMN). In a second step, three isoforms of NMN adenylyltransferase (NMNAT) can generate NAD^+^. To analyze the role of intracellular NAD^+^ (iNAD^+^) in M-CSF ± IL-10 driven P2Y_11_ receptor upregulation, we induced iNAD^+^ depletion through treatment with the NAMPT inhibitor FK-866 (36). NAMPT inhibition effectively prevented P2Y_11_ receptor upregulation (Figure 4). Extracellular NAD^+^ (eNAD^+^) has been shown to permeate the plasma membrane (37). eNAD^+^ thus increased iNAD^+^ contents and also replenished iNAD^+^ pools during FK-866 induced iNAD^+^ depletion (37). In accordance, we found that eNAD^+^ at least partially restored P2Y_11_ receptor upregulation during NAMPT inhibition (Figure 4).

NMN is the metabolite directly downstream of NAMPT and represents the immediate precursor to NAD^+^. NMN has recently been shown to be taken up by solute carrier family 12 member 8 (Slc12a8) (38). Supplementation with NMN turned out to be at least as effective as NAD^+^ in restoring P2Y_11_ expression during NAMPT inhibition with FK866 (Figure 4).

**Figure 5:**
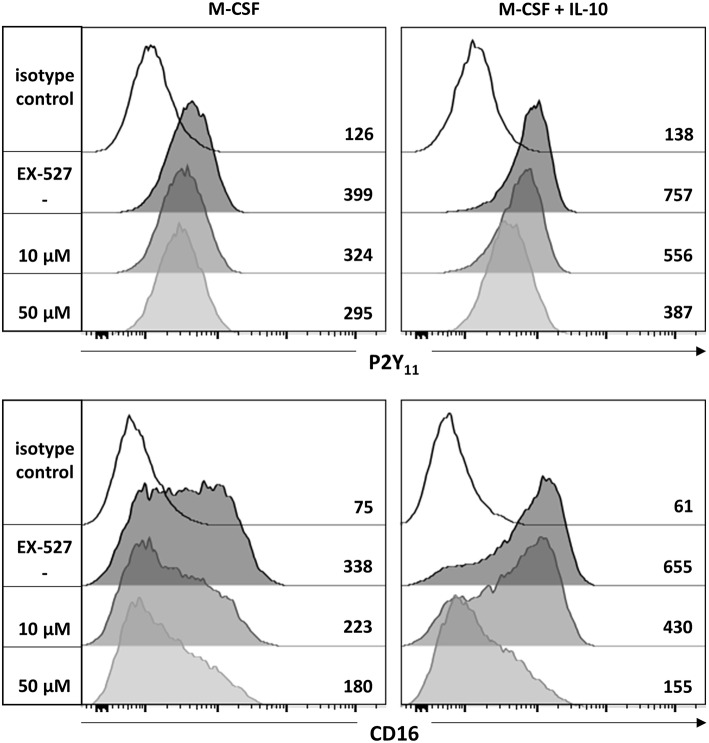
P2Y_11_ upregulation during M2c macrophage differentiation partially depends on SIRT1. CD16^−^CD14^+^ monocytes were left untreated (control) or stimulated overnight with M-CSF alone or in combination with IL-10 in the presence or absence of the SIRT1 inhibitor EX-527. P2Y_11_ receptor and CD16 surface expression were measured by flow cytometry. Numbers are mean fluorescence intensities.

The NAD^+^-dependent deacetylase Sirtuin 1 (SIRT1) has previously been implicated in the development of anti-inflammatory macrophages (39). To examine a potential involvement of SIRT1, we used the potent and selective SIRT1 inhibitor EX-527 (selisistat). We found that EX-527 prevented in a dose-dependent manner the upregulation of P2Y_11_ receptor as well as that of CD16 during M-CSF (M2) and M-CSF/IL-10 (M2c) induced polarization (Figure 5).

### Ectopic P2Y_11_ Receptor Promotes IL-8 Production

**Figure 6:**
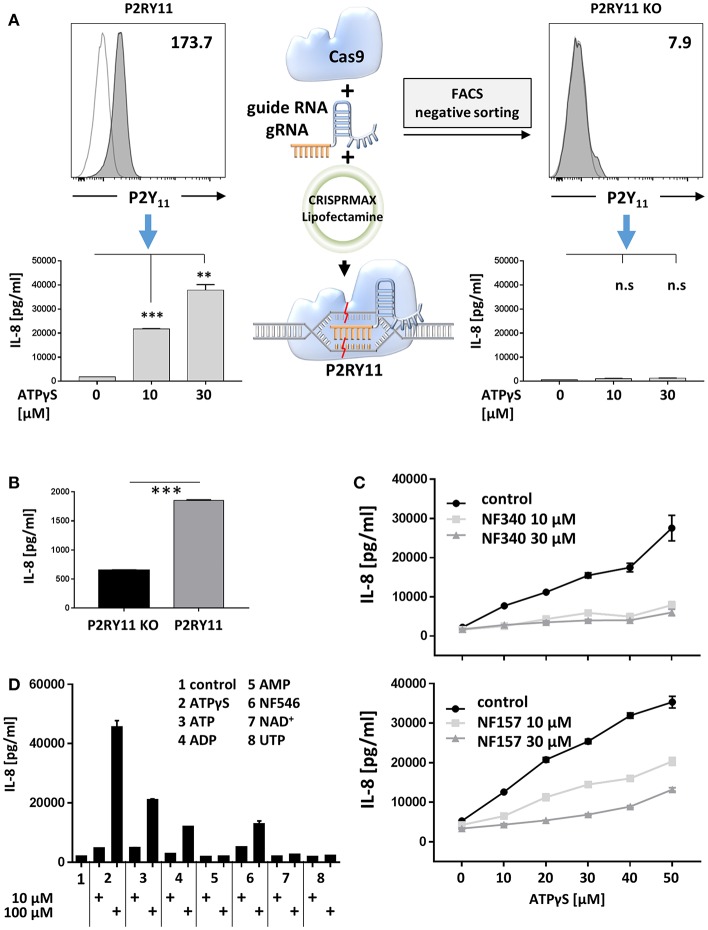
Ectopic P2Y_11_ drives IL-8 production in a recombinant glioma cell line. **(A)** P2Y_11_ surface expression was measured by flow cytometry in recombinant glioma cells (P2RY11) and in glioma cells subjected to CRISPR/Cas9-mediated P2RY11 knockdown (P2RY11 KO). P2Y_11_ agonist-induced IL-8 production was measured in supernatants of recombinant glioma cells and P2RY11 knockdown cells. Data are mean ± SEM (*n* = 3). Statistical analysis of the difference between unstimulated and agonist-treated P2RY11/P2RY11 KO cells was performed using One-Way ANOVA. ^**^p ≤ 0.01; ^***^p ≤ 0.001. **(B)** Spontaneous IL-8 production by recombinant glioma cells (P2RY11) and P2RY11-knockdown cells (P2RY11 KO). Statistical analysis of the difference between P2RY11 and P2RY11 KO in spontaneous IL-8 production was performed using one-tailed *t*-test. ^***^p ≤ 0.001. **(C)** IL-8 was measured in supernatants of recombinant glioma cells stimulated for 24 h with graded doses of the specific P2Y_11_ agonist in the presence or absence of the specific P2Y_11_ antagonists NF340, and NF157. **(D)** Identification of agonists that effectively induce IL-8 production in recombinant glioma cells.

To address P2Y_11_ receptor-driven cytokine responses, we first studied ectopic P2Y_11_ receptor in a recombinant system initially intended for drug discovery. In this cell system, P2RY11 is stably expressed in the glioma cell line 1321N1, a grade II human brain astrocytoma that proved to be an efficient GPCR expression system (40) and, above all, is naturally devoid of functional P2X and P2Y receptors (26). To obtain a control cell line, we performed targeted P2RY11 gene disruption using CRISPR/Cas9-edited knockdown, which resulted in complete loss of P2Y_11_ receptor surface expression (Figure 6A). Like many cancer cell lines, the 1321N1 glioma cell line spontaneously produced IL-8, which is a target of Ras signaling (41). Interestingly, in the absence of exogenous agonist, P2Y_11_ receptor-expressing cells produced significantly more IL-8 than their knockdown counterparts (Figure 6B). Moreover, P2Y_11_ receptor stimulation triggered in a dose-dependent manner the secretion of high levels of IL-8 in recombinant cells but not in knockdown cells (Figure 6A). Tumor necrosis factor- α (TNF-α), GM-CSF, CCL2 (MCP-1), and CXCL1 (GROα) were also measured but remained undetectable. The suramin analog NF340, which is a competitive antagonist and currently the most useful inhibitor at the P2Y_11_ receptor (1, 9), inhibited IL-8 production (Figure 6C). NF157, which is another suramin-derived competitive antagonist at the P2Y_11_ receptor (42) also inhibited IL-8 production, albeit somewhat less effectively (Figure 6C).

To identify agonists that effectively induce IL-8 production, we stimulated P2Y_11_ receptor-expressing cells with synthetic or natural adenine and uridine nucleotides at 10 and 100 μM. Consistent with the current view (1), ATPγS, ATP, and ADP but not AMP stimulated IL-8 production (Figure 6D). The non-nucleotide P2Y_11_ receptor agonist NF546 (9) also stimulated IL-8 production. In contrast, UTP, which has previously been postulated to be a P2Y_11_ agonist (43), failed to enhance IL-8 secretion. Moreover, NAD^+^, which has been considered an agonist at the human P2Y_11_ receptor (5), also turned out to be ineffective (Figure 6D).

### IL-8 Production Induced by ATPγS but Not by NAD^+^ Is Mediated by P2Y_11_ Receptor in M2c Macrophages

**Figure 7:**
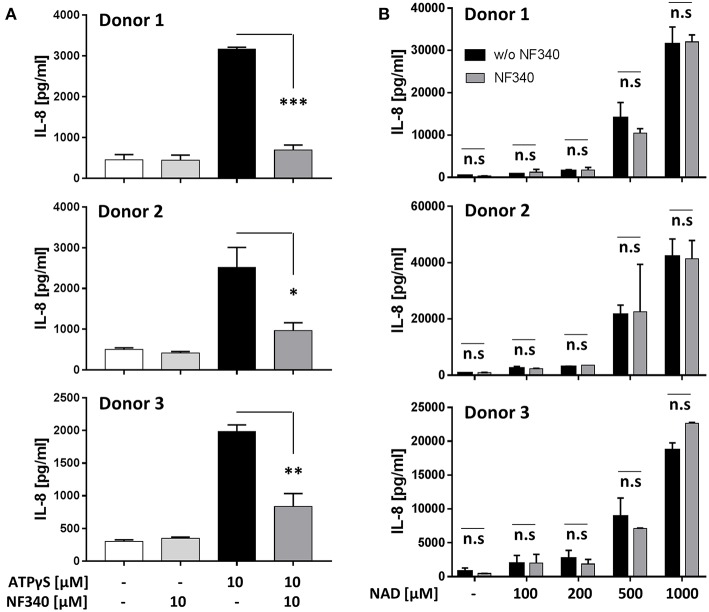
IL-8 production induced by ATPγS but not by NAD^+^ is mediated by P2Y_11_ receptor in M2c macrophages. **(A)** CD16^−^CD14^+^ monocytes were cultured for 5 d with M-CSF and IL-10 (M2c protocol). Cells were washed and replated at 50,000 cells per 0.1 mL. Cell were stimulated with specific P2Y_11_ agonist in the presence or absence of the specific P2Y_11_ antagonists NF340. After 24 h, IL-8 was measured in supernatants. Data are mean ± SEM (*n* = 3). Statistical analysis was performed using one-tailed *t*-test. ^*^p ≤ 0.05; ^**^p ≤ 0.01; ^***^p ≤ 0.001. **(B)** Day-5 M2c macrophages were washed, replated at 50,000 cells per 0.1 mL and then stimulated with graded doses of NAD^+^ in the presence or absence of the specific P2Y_11_ antagonist NF340. After 24 h, IL-8 was measured in supernatants.

Our findings in the recombinant system together with a previous report (9) indicated that IL-8 is a target of P2Y_11_ receptor signaling. We therefore measured IL-8 secretion from M2c macrophages after stimulation of native P2Y_11_ receptor. Similar to P2Y_11_ receptor-transfected glioma cells (Figure 6B), P2Y_11_ receptor-expressing M2c macrophages spontaneously produced substantial amounts of IL-8 (Figure 7). In three different donors, ATPγS induced a significant increase in IL-8 production that could be effectively inhibited with the P2Y_11_ receptor antagonist NF340 (91, 86, and 56%) (Figure 7A). eNAD^+^ at concentrations up to 200 μM had relatively little effect on IL-8 production. In contrast, eNAD^+^ at high concentrations (0.5 and 1 mM) induced strong IL-8 secretion from M2c macrophages, however these eNAD^+^-elicited responses were not significantly affected by NF340 (Figure 7B).

### Native P2Y_11_ Receptor Drives IL-8 Production in M2c Macrophages Predominantly via IKK/NFκB and MEK/ERK Pathways

**Figure 8:**
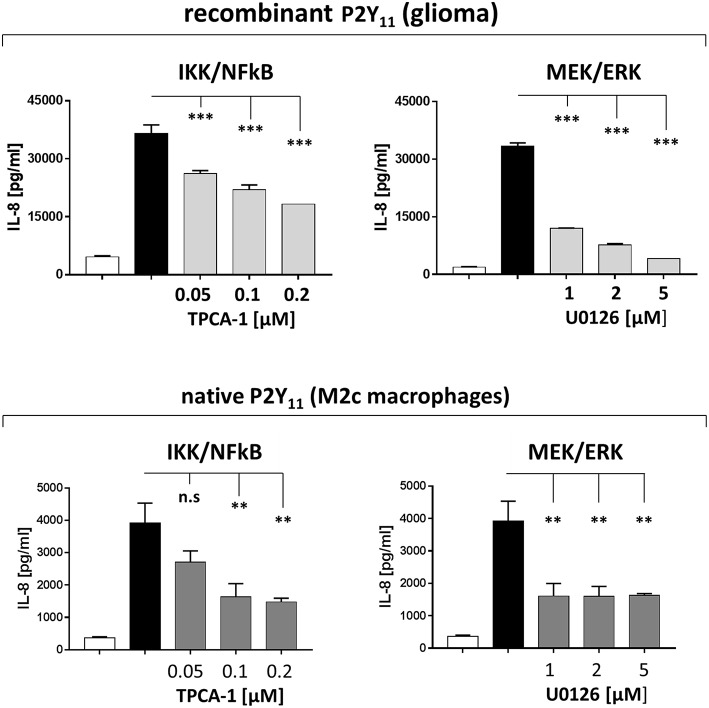
Native P2Y_11_ receptor drives IL-8 production in M2c macrophages predominantly via IKK and ERK. Day-5 M2c macrophages were washed, replated at 50,000 cells per 0.1 mL and then stimulated with specific P2Y_11_ agonist (ATPγS at 10 μM) in the presence or absence of inhibitors of signaling components. After 24 h, IL-8 was measured in supernatants. Data are mean ± SEM (*n* = 3). Statistical analysis of inhibitor effects was performed using One-Way ANOVA. ^**^p ≤ 0.01; ^***^p ≤ 0.001.

IL-8 is a transcriptional target of Ras signaling (41). Ras-mediated transcriptional upregulation of IL-8 has been shown to require the concurrent activation of the MEK/ERK and the IKK/NFκB effector pathways (41). P2Y_11_ receptor may also activate these pathways, since small GTPases of the Ras superfamily are known to be activated by GPCRs (44). Consistently, we found that inhibition of IKK (TPCA-1) and MEK (U0126) substantially inhibited P2Y_11_ receptor-driven IL-8 production, both in the recombinant glioma cell line and in primary M2c macrophages (Figure 8).

## Discussion

This work demonstrates that metabolic reprogramming of human macrophages during IL-10 driven M2c differentiation involves the strong upregulation of the metabotropic P2Y_11_ receptor. It is the first example of P2Y_11_ receptor regulation at the protein level. We also unveil the critical role of an AMPK-NAMPT-NAD^+^ signaling axis in IL-10 induced P2Y_11_ receptor upregulation. Specifically, we identify NAD^+^ as a macrophage metabolite promoting P2Y_11_ receptor expression rather than being itself a P2Y_11_ agonist in this cell type. Previous work had proposed NAD^+^ to be a P2Y_11_ receptor ligand in human granulocytes mediating the sustained increase of intracellular Ca^2+^ required for functional activation (5). The specific P2Y_11_ antagonist NF340 has not yet been available at that time. Instead, NF157 was used to inhibit the NAD^+^-induced Ca^2+^ response. However, NF157 is non-selective over P2X_1_ receptors and has low (P2X_2_, P2X_3_) to moderate (P2X_4_, P2X_7_) selectivity over other P2X subtypes (1, 42). Its activity at other P2X and P2Y subtypes has not been reported (1). In addition, potential stimulatory effects of NAD^+^ on P2Y_11_ receptor surface expression could not be explored because of the lack of specific antibodies (1, 2).

**Figure 9:**
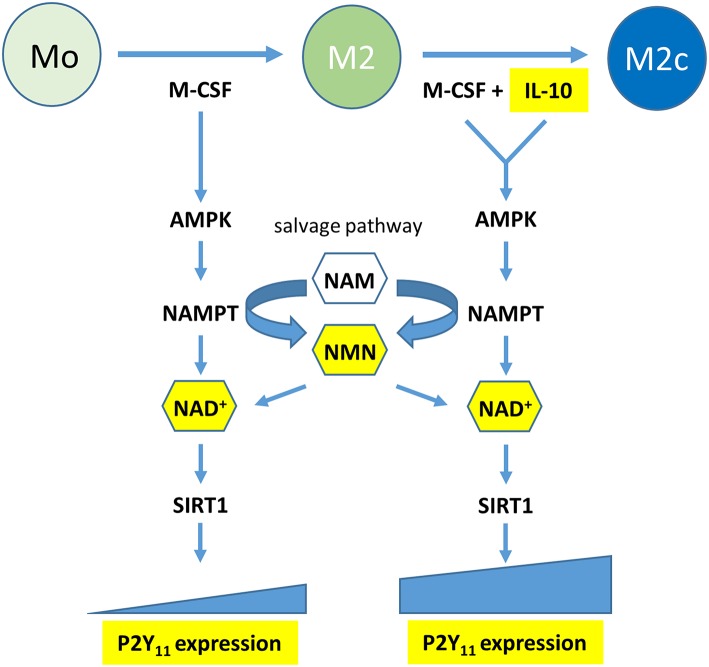
Graphical summary: the metabolic reprogramming during macrophage polarization induced by M-CSF (M2) and reinforced by IL-10 (M2c) leads to P2Y_11_ receptor upregulation. Metabolic reprogramming involves activation of a signaling axis comprising AMP-activated kinase (AMPK), nicotinamide phosphoribosyltransferase (NAMPT), and NAD^+^. NAMPT is the rate-limiting enzyme of the NAD^+^ salvage pathway, which converts nicotinamide (NAM) into nicotinamide mononucleotide (NMN). NAD^+^ acts as macrophage metabolite that drives P2Y_11_ receptor expression, possibly via the NAD^+^-dependent deactylase SIRT1.

Functional P2Y_11_ receptor expression has also been documented in moDCs (8–11), however, high agonist dosages were sometimes required to elicit P2Y_11_ receptor responses or agonist effects could not be controlled reliably due to the lack of specific P2Y_11_ receptor inhibitors. Our present work demonstrates that IL-4, which is used to generate moDCs (16, 17), attenuates P2Y_11_ expression, resulting in relatively low levels of P2Y_11_ receptor protein on the surface of moDCs. In contrast, IL-10, which prevents moDC development, and favors macrophage differentiation (24), was the dominant driving force that promoted P2Y_11_ receptor upregulation even in the presence of IL-4. The anti-inflammatory effects of IL-10 are known to be mediated by metabolic reprogramming of macrophages, which includes activation of AMPK (18, 21). AMPK in turn increases the levels of intracellular NAD^+^ by engaging the salvage pathway of NAD^+^ biosynthesis (3, 22). Although the term “salvage” implies engagement only in case of emergency, mammalian cells predominantly rely on the NAMPT-dependent pathway for NAD^+^ biosynthesis (45). Accordingly, we found that inhibition of AMPK and NAMPT, the rate-limiting enzyme of the salvage pathway, prevented P2Y_11_ receptor upregulation during M2c macrophage differentiation. Intriguingly, NAD^+^ as well as its immediate precursor NMN, which are both known to permeate the plasma membrane and replenish intracellular pools during FK-866 induced depletion (37, 38), could restore P2Y_11_ receptor upregulation during NAMPT inhibition. It cannot be excluded that such effects of eNAD^+^ have contributed to P2Y_11_ receptor-induced responses observed in earlier studies (5). Collectively, our current findings shift the view from NAD^+^ as an agonist to NAD^+^ as a metabolite that promotes P2Y_11_ receptor expression during AMPK-NAMPT-driven metabolic reprogramming. NAD^+^ may accomplish this at least in part by activating SIRT1 (Figure 9).

In addition to its role in cancer prevention, p53 participates in metabolic checkpoints (23), which are also known to be critical for macrophage differentiation (18–21). Macrophages have endogenous p53 activity, which may even increase during M2 polarization (31). We found that p53 stabilization induced by the MDM2 inhibitor nutlin-3a caused a further increase of P2Y_11_ expression in M2c macrophages. This is of particular interest, because the promoter region of human P2RY11 was previously reported to contain three p53 non-canonical response elements (32). In addition to protein-protein interactions, activated tetrameric p53 binds to promoter regions of target genes via DNA response elements (REs) and modulates gene transcription. A large number of p53 REs can be predicted to be functional even if the RE motif is incomplete. The classification of REs (grade 1 to 5) reflects associated functional scores. The majority of REs that are likely to be functional are in the grade 2 category. Although they must be expected to be poorly responsive on their own, these REs could participate in the regulation of gene expression if the context is appropriate. Besides canonical, full site responsive elements (REs), non-canonical half sites, and ¾ (3Q) sites have been identified that can mediate p53-dependent responsiveness of associated coding sequences. Among the three non-canonical REs detected in the promoter region of human P2RY11 (32) is a grade 1 RE considered unlikely to be functional, but also one grade 2 and one grade 3 REs, which may indeed have played a role in the nutlin-3a induced upregulation of P2Y_11_ receptor.

Using our recombinant system, we clearly identified IL-8 as a target of P2Y_11_ receptor signaling. IL-8 production induced by selective agonist could be inhibited by specific antagonists. Moreover, CRISPR/Cas9-mediated receptor knockdown abolished agonist-induced IL-8 production. Interestingly, the recombinant glioma host cell line expressing P2Y_11_ receptor displayed higher levels of spontaneous IL-8 production (i.e., in the absence of agonist) compared to their knockdown counterparts. Since IL-8 is also a known target of Ras signaling, our findings suggested that P2Y_11_ receptor stimulation enhances the Ras-mediated activation of the ERK and IKK effector pathways (41). In accordance with this concept, we found that ERK and IKK inhibitors prevented P2Y_11_ receptor-driven IL-8 secretion.

Increased IL-8 expression and signaling has been characterized in tumor-associated macrophages, suggesting that IL-8 may act as a significant regulatory factor within the tumor microenvironment (46). In addition, stress, and drug-enhanced IL-8 signaling has been shown to contribute to the development of chemotherapy resistance in cancer cells. As an ATP receptor, P2Y_11_ may also be involved in such therapy-induced effects, because a wide range of chemotherapeutic agents causes the release of ATP into the extracellular space as they induce tumor cell death (47). According to our current observations, triggering of the P2Y_11_ receptor by therapy-induced extracellular ATP would then stimulate IL-8 secretion from M2c macrophages, facilitating chemoresistance, and tumor progression.

As with ATP, substantial amounts of the dinucleotide NAD^+^ can also arise in the microenvironment of stressed and damaged cells (48). Our study unexpectedly showed a strong stimulatory effect of eNAD^+^ on IL-8 production by M2c macrophages, a response that appeared to be P2Y_11_ receptor-independent. However, this eNAD^+^ effect occurred only at high doses (≥500 μM), i.e., 50-fold higher than the dose of ATPγS (10 μM). When the two compounds were tested side-by-side in the recombinant system, only ATPγS displayed agonist activity. NAD^+^ has recently been shown to govern a secretory response associated with aging and cancer that includes IL-8 production (4). The overall effect of this secretory response is considered detrimental in tumors because it promotes hallmarks of cancer, including tumor growth and angiogenesis.

In conclusion, we show for the first time regulation of human P2Y_11_ receptor at the protein level and associate it with the differentiation of M2c macrophages in response to IL-10. P2Y_11_ receptor upregulation occurs during IL-10 induced metabolic reprogramming and involves an AMPK-NAMPT-NAD^+^ signaling axis. M2c macrophages coexpress PD-L1, and p53 activation increases the expression of both, P2Y_11_ receptor and PD-L1, identifying this IL-8 producing macrophage subset as an important target of checkpoint blockade immunotherapy. The anti-inflammatory M2c subset has been implicated in the clearance of early apoptotic cells (29), which is also essential for endothelial surveillance. Successful targeting and elimination of this population during checkpoint inhibition therapy may therefore increase the risk of vasculitis, a rare but severe side effect of this type of immunotherapy (49).

## Data Availability

The datasets generated for this study are available on request to the corresponding author.

## Ethics Statement

Inclusion of healthy donors was approved by the local institutional review board (EK Nr: 1087/2018). Buffy coats were obtained after written informed consent and provided by the Central Institute for Blood Transfusion (Innsbruck, Austria).

## Author Contributions

GG and MT designed and supervised the study. WN acquired blood samples and performed cell enrichment as well as quality control. HG, GD, and AD performed monocyte isolation, differentiation, and stimulation as well as propagation of cell lines. AR carried out cytokine measurements. GG conducted CRISPR/Cas9 knockdown studies and operated the Canto II flow cytometer. GG, JT, and MT analyzed and interpreted data. GG prepared graphs. MT and GG wrote and revised the manuscript.

### Conflict of Interest Statement

The authors declare that the research was conducted in the absence of any commercial or financial relationships that could be construed as a potential conflict of interest.
