# Supplementary material for: The Human G Protein-Coupled ATP Receptor P2Y11 Is Associated With IL-10 Driven Macrophage Differentiation
Source: Front Immunol. 2019 Aug 9;10:1870. doi: 10.3389/fimmu.2019.01870 (PMC6695557; doi:10.3389/fimmu.2019.01870)
Supplement: Supplementary file 1 [file Data_Sheet_1.PDF]

# The human G protein-coupled ATP receptor $P2Y_{11}$ is associated with IL-10 driven macrophage differentiation

Georg Gruenbacher[1], Hubert Gander[1], Andrea Rahm[1], Gabriele Dobler[1], Astrid Drasche[2], Jakob Troppmair[2], Walter Nussbaumer[3], Martin Thurnher[1]

**Running title:** $P2Y_{11}$ in macrophage differentiation

**Author affiliations:**

[1]Immunotherapy Unit, Department of Urology, Medical University of Innsbruck, 6020 Innsbruck, Austria – EUROPE;

[2]Daniel Swarovski Research Laboratory, Department of Visceral-, Transplant- and Thoracic Surgery, Medical University of Innsbruck, 6020 Innsbruck, Austria – EUROPE

[3]Central Institute for Blood Transfusion and Immunology, Medical University Hospital Innsbruck, Innsbruck, Austria - EUROPE

## Legend to Figure S1

### Phagocytosis of latex beads by M2c macrophages

Latex beads (diameter, 4 µm; Thermo Fisher Scientific) were centrifuged (1 min at 100g) onto adherent day-5 M2c macrophages at a ratio of 10:1 to synchronize binding and internalization. After 1 h at 37 °C, nonadherent beads were removed with cold PBS and cells were immediately photographed by phase contrast microscopy (Olympus CK2, magnification: 200-fold).

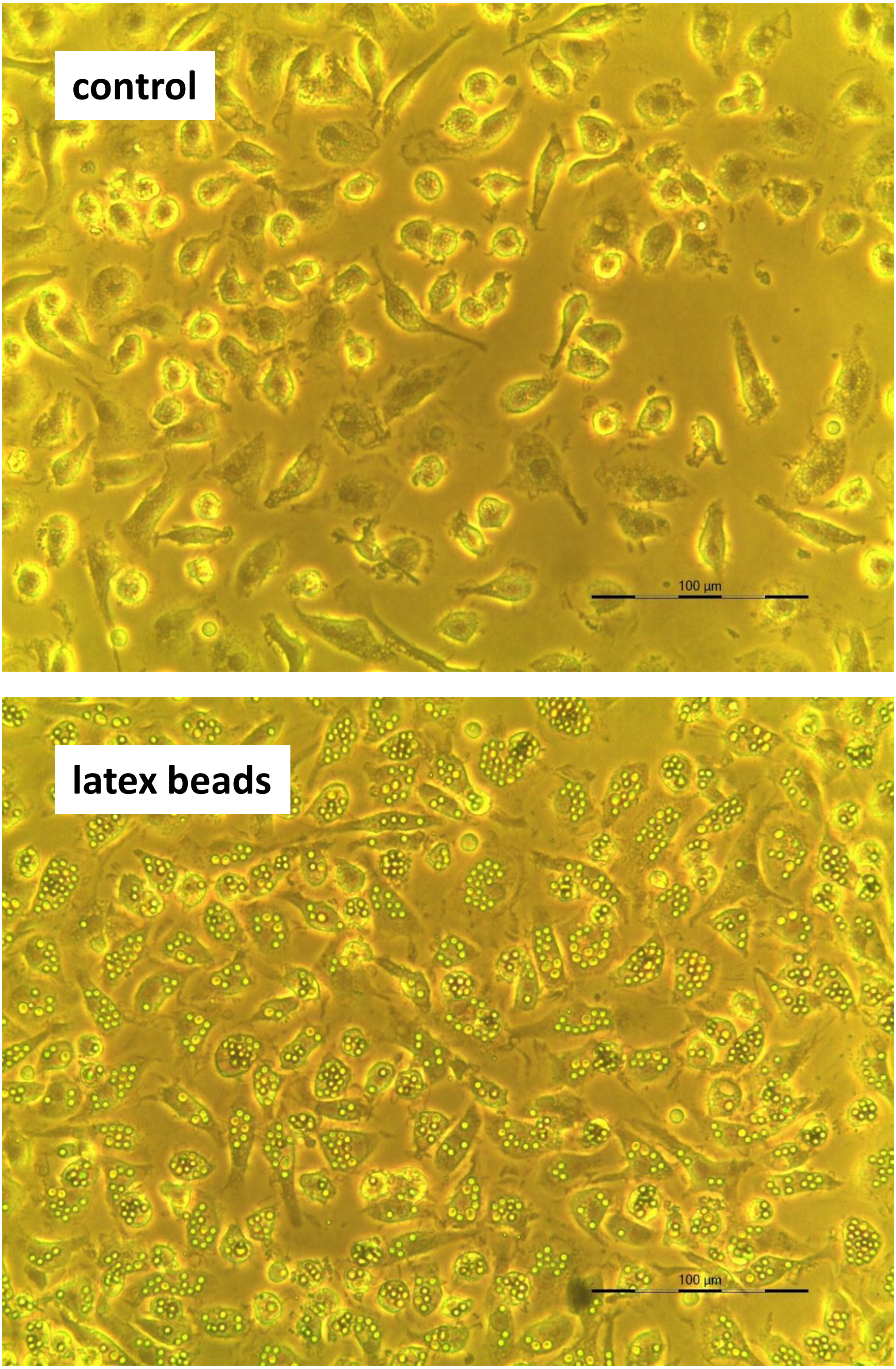


Figure S1
